# From sequence to function: Insights from natural variation in budding yeasts^☆^

**DOI:** 10.1016/j.bbagen.2011.02.004

**Published:** 2011-10

**Authors:** Conrad A. Nieduszynski, Gianni Liti

**Affiliations:** Centre for Genetics and Genomics, University of Nottingham, Queen's Medical Centre, Nottingham, UK

**Keywords:** *Saccharomyces cerevisiae*, Forward genomics, Reverse genomics, Functional analysis, Quantitative trait locus, Comparative genomics

## Abstract

**Background:**

Natural variation offers a powerful approach for assigning function to DNA sequence—a pressing challenge in the age of high throughput sequencing technologies.

**Scope of Review:**

Here we review comparative genomic approaches that are bridging the sequence–function and genotype–phenotype gaps. Reverse genomic approaches aim to analyse sequence to assign function, whereas forward genomic approaches start from a phenotype and aim to identify the underlying genotype responsible.

**Major Conclusions:**

Comparative genomic approaches, pioneered in budding yeasts, have resulted in dramatic improvements in our understanding of the function of both genes and regulatory sequences. Analogous studies in other systems, including humans, demonstrate the ubiquity of comparative genomic approaches. Recently, forward genomic approaches, exploiting natural variation within yeast populations, have started to offer powerful insights into how genotype influences phenotype and even the ability to predict phenotypes.

**General Significance:**

Comparative genomic experiments are defining the fundamental rules that govern complex traits in natural populations from yeast to humans.

This article is part of a Special Issue entitled Systems Biology of Microorganisms.

## Research Highlights

► A major challenge in biology is the assigning of function to DNA sequence. ► We review the power of comparative genomics and natural variation. ► We contrast forward and reverse genomic approaches. ► We present a case study of telomere biology as a complex phenotype.

## 1 Introduction

**Fig. 1:**
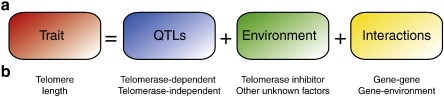
The makeup of a complex trait. (a) Complex traits are regulated at the genetic level by multiple quantitative trait loci (QTLs), the environment and interactions between them (Adapted from Fig. 1 in reference 88). (b) Telomere length, as an example of a complex trait, consists of the number of repeats maintained at the end of the chromosomes. This is regulated by genes that modulate the activity of telomerase (e.g. *YKU80*) and genes whose activity is telomerase-independent (e.g. *ELG1*). Telomere length is also determined by environmental factors (including inhibitors of telomerase [85]) although this is yet to be comprehensively screened. Finally, interactions between genes (e.g. *YKU70* and *YKU80*[83]) and between genes and the environment [85] contribute to telomere length homeostasis.

A fundamental objective in modern biology is the assignment of function to DNA sequence. This has been heightened by the completion of major genome sequencing projects and the advent of high throughput sequencing technologies. The accelerating output from genome sequencing projects provides a wealth of data for tackling fundamental biological questions and emphasises the importance of assigning function to DNA. A related and potentially more challenging objective is to understand the link between phenotype and genotype; this difficulty is in part due to the complexity of a trait. Phenotypes are determined at several levels, at multiple loci, the environment and interactions between the loci and the environment (Fig. 1a; [1]). Most phenotypes vary quantitatively across natural populations allowing insight to be gained from their study.

The unicellular eukaryotic *Saccharomyces cerevisiae* has provided a formidable model to bridge the sequence to function gap. The *Saccharomyces* genome database [2] has evolved from cataloguing open reading frames (ORFs) to an ever-increasing number of features and associated phenotypes, making it the organism with the best-characterised genome. Many of these annotations were gathered from systematic studies using the laboratory strain S288c or close relatives with shared ancestry.

In the past decade we have witnessed an explosion in the number of studies that use comparative genomic analyses, between both strains and species, to precisely dissect biological processes. Here, we focus on how these studies have aided the assignment of function to sequence and recently started to link phenotype and genotype. Although these approaches are still in their infancy we discuss their potential.

**Fig. 2:**
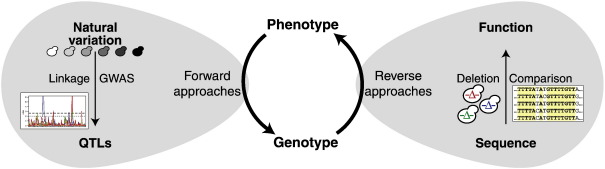
Different routes from genotype to phenotype. Complementary forward and reverse genetic approaches to understanding cellular traits.

There are two ways to exploit natural variation: reverse and forward genomics (Fig. 2). Reverse genomics consists of identifying a specific sequence, either coding or non-coding, and try to assign a function using sequence comparison or functional analysis. Conversely, forward genomic approaches start from a trait and without any *a priori* hypothesis seek to dissect the underlying genetic mechanism.

## 2 Comparative ‘reverse’ genomics

### 2.1 Background: from classical reverse genetics to reverse genomics

Reverse genetics starts from a gene (or sequence) and aims to determine the function of that gene. Examples of reverse genetic approaches in yeasts have been reviewed elsewhere [3]. The advent of whole genome sequencing allowed the scaling up of these approaches to ‘reverse genomics.’ Here we describe some of what has been learnt from reverse genomic approaches, focusing on comparative genomic studies.

### 2.2 Sequence analysis

Huge quantities of genome sequence have favoured automated approaches to assigning function. Computational methods have allowed the broad classification of sequence function (for example, the identification of protein-coding sequences) and the annotation of genome sequences. The availability of genome sequences from several closely related species has allowed comparative approaches that have greatly aided in the annotation of genomes and the assignment of function to sequence (Table 1). Yeast has played a pivotal and early role in these comparative genomic studies.

The power of comparative genomic approaches is the ability to select different and appropriate degrees of evolutionary divergence to answer specific questions. The *Hemiascomycetes* yeast species have proved well suited to this analysis due to the rich representation of species across a wide range of evolutionary divergences and their compact genomes [4]. In addition many of these species are themselves biologically important, both industrially (e.g. *Kluyveromyces lactis* and *Pichia stipitis*) and medically (e.g. *Candida albicans*). Phylogenetic comparison of sequence offers the opportunity to improve gene annotation and map regulatory regions as well as to elucidate the mechanisms of genome evolution [4]. Two different strategies marked the start of the yeast comparative genomics era. One was to sequence multiple closely related *Saccharomyces* species [5,6]. Earlier work with this set of *Saccharomyces* species showed the power of comparing closely related species to dissect the mechanisms of genome evolution and reproductive isolation [7,8]. The other strategy, undertaken by the French consortium *Génolevures*, sequenced and compared the genome of highly diverged *Hemiascomycetes* yeast species that span a much broader evolutionary divergence [9,10]. Comparison of the genomes from divergent yeast species has provided insight into mechanisms of genome evolution, including the expansion and contraction of gene families as individual species adapt to particular lifestyles [11,12]. In addition, genome sequencing of six *Candida* species has given insight into their pathogenicity [13]. More recently, population genomics data from sequencing [14] and microarray analysis [15] of multiple individuals of the same species have added a third level of sequence divergence.

The initial annotation of protein coding sequences in *S. cerevisiae* was aided by the scarcity of spliced genes; hence the majority of genes could be identified as open reading frames (ORFs, defined as greater than 99 codons). The availability of genome sequences from other *Saccharomyces* species allowed the refinement of these annotations based upon the pattern of sequence conservation [5,6,16,17]. This involved the elimination of ~ 500 spurious ORFs, identification of 43 additional small ORFs (50–99 codons), refinement of initiation and termination codons and the identification of new introns. In total ~ 15% of the ORF annotations were adjusted. Many of these annotation improvements have made further analysis of these genes possible; for example many functional studies use gene tags, an approach that requires accurate knowledge of the start and stop codons.

The sequencing of multiple individuals of the same species allows the identification of single nucleotide polymorphisms (SNPs) and presents the challenge of distinguishing deleterious from neutral SNPs. Computational approaches (e.g. SIFT [18]) based upon the principle that a site conserved between species is less likely to tolerate polymorphisms allow the prediction of which SNPs will be deleterious [19]. These methods also take into account the nature of the amino acid change and known protein domain information. Application of these methods to the genome sequence from three *S. cerevisiae* strains predicted that 12% of coding and 7% of noncoding SNPs are deleterious [20]. Population genomics data have revealed the degree of selection and proportion of deleterious polymorphisms (both SNPs and indels) on a larger scale [14,21].

**Fig. 3:**
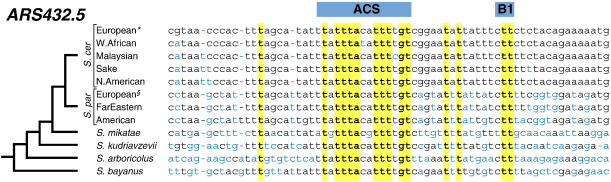
The phylogenetic footprint at a budding yeast replication origin identifies the functional protein-binding motif. Whole genome alignments from *sensu stricto* species can be analysed to identify phylogentically conserved motifs. Shown, is a region from an alignment spanning the replication origin *ARS432.5*[89]. This identifies a conserved sequence element that matches the motif (called the ACS) found at *S. cerevisiae* replication origins. Mutation of this conserved motif was found to abolish origin activity [23]. A second origin element (called the B1) is also found to be phylogenetically conserved [90]. Bases conserved across all strains and species are shown in bold and highlighted in yellow, bases that differ from the European *S. cerevisiae* sequence are shown in blue. The alignment includes the five clean lineages of *S. cerevisiae* (European: DBVPG6765; West African: DBVPG6044; Malaysian: UWOPS03.461.4, Sake: Y12; and North American: YPS128), three clean lineages of *S. paradoxus* (European: CBS432; Far Eastern: N-44; American: YPS138), *S. mikatae*, *S kudriavzevii*, *S. arboricolus* and *S. bayanus*[5,6,14,91]. *The sequence of *ARS432.5* in the European *S. cerevisiae* strain is the same as the reference genome (S288c). ^§^CBS432 is the *S. paradoxus* reference strain. Note the phylogenetic tree represents the topology of the *sensu stricto* group, but branch lengths are not drawn to scale.

The identification of functional DNA regulatory sequences is more challenging than coding regions, despite the fact that they are generally described by a sequence motif. This is a consequence of how short the motifs are, that they typically tolerate some sequence variation and (in contrast to genes) that they do not have a clearly defined start or stop. In addition, the repertoire of functional regulatory sequences is frequently only a small fraction of the total occurrences of a particular motif in the genome. These motifs are protein bound (for example by a transcription factor) and therefore the evolution of the sequence is restrained, resulting in a ‘phylogenetic footprint’ (Fig. 3). Comparative genomic approaches have allowed the identification of conserved occurrences of sequence motifs [6,17]. Combining comparative genomics with experimental data for protein binding sites has allowed the identification with base pair resolution of functional sequences bound by transcription factors [22] and DNA replication proteins [23,24]. Analysis of recently available population genome sequence data [14] has facilitated improvements in the assignment of function to sequences, including the identification of novel genes [14], intron splice sites [25] and regulatory sequences [26]. Therefore, comparisons across a range of evolutionary divergences have helped bridge the sequence to function gap, from pinpointing DNA sequence elements [5,6] through to identifying virulence factors [13].

### 2.3 Functional analysis—lessons from gene deletion studies

The functional analysis of a gene or sequence frequently involves the generation of a deletion or mutant, followed by phenotypic analysis to gain functional insight. This type of analysis can blur the distinction between reverse and forward approaches, for example, when applied as a phenotype screen to a library of random mutants. At the genomic level the deletion collections targeted specific gene sequences and current approaches normally phenotype the entire collections of mutants either individually or as a pool.

The *S. cerevisiae* gene deletion collection was the first such genome-wide deletion (or gene inactivation) collection and has played a key role in assigning gene function. Large scale studies using this collection have included gene dispensability [27], synthetic lethality studies [28], haploinsufficiency under competitive growth conditions [29] and chemical environmental genetics [30,31]. The gene deletion collections have frequently confirmed results from classical genetic experiments and have allowed comprehensive testing of the gene repertoire. The deletion collection was built on the BY strain background, a derivative of S288c, and different versions exist (e.g. haploid, diploid homozygous or heterozygous deletion). The deletion collection revealed that 1033 genes are essential in the *S. cerevisiae* strain S288c. It has proved challenging to study these essential genes with the deletion collection, but three general approaches have been developed. One strategy uses heterozygous diploids to reduce gene dosage, but most strains show no obvious phenotype [32]. A second approach generated hypomorphic alleles, for the majority of yeast essential genes, by destabilising and therefore reducing the steady state levels of the mRNA [33]. A final approach involves inducible gene inactivation either by transcriptional shut-off [34] or conditional protein destabilisation [35,36]. Several of these methodologies are directly applicable to other model systems. These approaches span a range of levels of gene inactivation and together they have contributed to elucidating the function of essential yeast genes.

A nearly complete deletion collection is now available for the distantly related yeast model *Schizosaccharomyces pombe* [37]. This offers the opportunity for comparative analysis to determine the generality of the observed phenotypes and further expand our knowledge of particular genes. At a fundamental level, the large majority (83%) of *S. cerevisiae* and *S. pombe* single copy orthologs share dispensability. Recently, a collection of 674 guided gene deletions from the yeast pathogen *C. albicans* has been screened for infectivity, morphogenesis and proliferation [38]. Interestingly, these screens identified several mutants that were defective for infectivity without affecting colony morphology or proliferation—phenotypes that have been proposed to be correlated.

Further complexity in the regulation of gene dispensability was recently reported by screening for essential genes in a different *S. cerevisiae* strain background [39]. The authors generated 5100 gene deletions in another laboratory strain (∑ 1278b). This strain shares ancestry with S288c [40] and the sequence of half of the orthologous genes are identical [39]. Despite being closely related, Dowell et al. found that 44 genes are essential in ∑ 1278b and not in S288c and 13 vice versa. The majority of these genetic background conditional essential genes are complexly regulated by a large number of modifiers (> 3). It will be important to understand the regulation of conditionally essential genes and to determine if there are shared modifiers. Modifiers need not be limited to genes; for example, even chromosome structure can determine gene essentiality as previously determined for the deletion of a histone gene [41]. This mechanism involved a gene dosage compensation by gene amplification where the presence of a retrotransposon (Ty1) mediated the formation of an extra circular chromosome.

Rapid advances in understanding gene function have been possible using the *S. cerevisiae* gene deletion collections. Many of the approaches that have been successfully used in *S. cerevisiae* can now be applied to the deletion collections in other yeast species. In addition, the relative ease with which the gene deletion cassettes can be transferred between closely related isolates (of the same species) [39] will allow the establishment of further deletion collections and analysis of phenotypic variation within populations. Such comparative approaches, potentially coupled with ‘forward’ genomics, can illustrate how genetic variants determine complex phenotypes including in human diseases.

## 3 Comparative ‘forward’ genomics

### 3.1 Background: from forward genetics to genomics

Classical forward genetic studies were successful in mapping mutations that affected a trait (e.g. radiation sensitivity). These studies involved first screening a randomly mutagenised strain for a specific phenotype followed by genetic analysis to identify the responsible allele. The advent of high throughput sequencing technologies has aided in the identification of the alleles and allowed the application of these approaches to organisms that are not genetically amenable. Comparative forward genomic approaches use similar principles, but instead of creating artificial mutants, they exploit natural phenotypic variation. Phenotypes vary quantitatively in natural populations as a result of an underling complex polygenic architecture.

### 3.2 Yeast as a model for quantitative genetics

Most yeast genetics studies have used the laboratory strain S288c or its derivatives. The difference in genetic background often resulted in incongruence that complicated the interpretation of results (for examples see [41–43]). These quantitative differences are now recognised as a major resource and have permitted the foundation of yeast forward genomic approaches. Forward genomics, in the budding yeast, started relatively late compared to other genetic model systems, due to the strength of classical reverse genetics that captured significant research effort. However, yeast has all the key features required to successfully apply forward genomic approaches. In fact, *S. cerevisiae* (and relatives) offer a powerful platform for forward genomics, including established classical genetic techniques that allow control of the sexual life cycle by crossing different strains, rapid isolation of segregants, high recombination rates that allow high resolution gene mapping, and a small genome size that facilitates easy and inexpensive genotyping. Recently there has been increased appreciation of the opportunities offered by the natural variation in either wild or fermentation-associated isolates and thousands of isolates have been described. Recently, a large collection of over 50 *S. cerevisiae* and *S. paradoxus* strains that sample the species variation and are amenable for genetic studies has been generated [44]. Therefore, despite being a latecomer to this field, *S. cerevisiae* is now a leading model for dissecting the cause of heritable variation.

There are two approaches to mapping quantitative trait loci (QTLs) [1]. Linkage analysis uses designed crosses between individuals with diverged phenotypes or sibling family studies. In contrast, association mapping utilises natural populations and historical recombination. Both approaches map functional polymorphisms based upon their linkage with known polymorphic markers and benefit from knowledge of the population structure for an effective experimental design.

### 3.3 Linkage analysis

Two groundbreaking papers established budding yeast as a model for linkage analysis by dissecting the complex architecture of high temperature growth [45] and variation in the abundance of gene transcripts [46]. Yeast linkage analyses using a small sample size (typically ~ 100 individuals) allow the mapping of QTLs that have high or intermediate effect [47] but only within a large interval (for example, within 10–50 genes). Linked QTLs have frequently been observed and these are particularly difficult to identify using classical linkage analysis [48,49]. Advances in high throughput genotyping, sequencing and phenotyping have permitted expansion of this approach. The addition of more segregants allows the mapping of QTLs with more modest effect as well as narrowing of the mapped interval. Working with yeast, allows the use of reverse genetic techniques to measure the phenotypic effect of individual SNP [50,51]. Recently, the problem of detecting QTLs with a small size effect has been partially solved by analysing segregants as large pools rather than individually [52,53]. This approach is based upon subjecting millions of pooled segregants to a selection for a phenotype and then analysing genome-wide changes in allele frequency within the population.

Several large effect QTLs have also been characterised, such as the *RAD5* allele [54] and the *CYS4* allele [55] in the *S. cerevisiae* wine strains RM11 and M22 respectively, that show a pattern of inheritance almost analogous to a Mendelian trait. These variants appear to be unaffected by the genetic context of the cross [56] and the responsible polymorphisms include non-synonymous substitutions, frameshifts, premature stop codons and structural variants. In contrast, many weaker QTLs have been found to be dependent on the genetic context [56,57]—i.e. the QTL can be detected in one cross combination but not in another. This dependency may result from interactions within the genetic background (e.g. strain specific modifiers). A consequence of this is that detection of a specific QTL can depend upon the strains analysed. One set of segregants, the BY (S288c derivative) × RM11 (vineyard isolate) series, has been used multiple times to investigate a broad range of phenotypes (reviewed in [58]). These studies revealed the presence of pleiotropic QTLs (hotspots). It remains to be determined whether these pleiotropic QTLs represent master regulators of multiple phenotypes or whether they arise from the similarity of the phenotypes examined.

The QTL mapping approaches discussed above currently map to genomic intervals containing multiple genes. Reciprocal hemizygosity offers a rapid tool to determine the gene responsible for the phenotype [48]. This approach compares phenotypes in F1 hybrids where either one allele or the other is deleted—i.e. the strains compared are isogenic except for the single allele being investigated. Computation methods (e.g. SIFT discussed above) then offer the possibility to predict the causative nucleotide polymorphism. Therefore, together these methods can reveal how genotype determines phenotype.

### 3.4 Experimental evolution and novel genes

Experimental evolution can give insight into the role played by particular genes in a phenotype. In this approach, yeast strains are exposed to long-term adaptive conditions during which beneficial genetic changes (adaptive mutations) are fixed in the population. Adaptive mutations have been mapped using bulk segregant analysis [59]. More recently, sequencing technologies have been used to map the adaptive changes. For example, mutations involved in adaption to low glucose, high salt, and limiting sulphate have been identified [60,61]. Studies that investigate natural isolates with different metabolic strategies (for example [62]) have the potential to determine whether common or different strategies are employed to adapt to the same environment. Romano et al. demonstrate that adaption to alkali stress targets different alleles to those varying in natural populations, demonstrating that evolution has found different solutions to the same challenge [63]. Combining experimental evolution with QTL analysis has the potential to give a broader picture of the pathways involved in a particular trait.

Forward genomic approaches that exploit natural variation have also been fruitful in characterising phenotypes with a simpler inheritance pattern. A recent study investigated the ability of some natural isolates of the *Saccharomyces sensu stricto* complex to ferment xylose [64]. Bulk segregant and microarray analyses linked this trait to a novel subtelomeric putative xylitol dehydrogenase (*XDH1*, also characterised in the wine strain EC1118 [65]). Another variable trait recently dissected is biotin biosynthesis. Two subtelomeric accessory genes (*BIO1* and *BIO6*), identified in a set of Sake strains, confer the ability to synthesise biotin [66]. These studies emphasise the important contribution of subtelomeres to phenotypic variation [67] and underscore the importance of sequencing strategies to complete the yeast gene repertoire [14,65].

### 3.5 Genome wide associations studies

Although yeast is now a prominent model for QTL mapping, there are no examples of genome wide associations studies (GWAS). Successful GWAS required natural populations with several features: high allele frequencies, rapid linkage disequilibrium decay and absence of population structure (uneven degrees of relatedness) [68,69]. The genomics survey revealed a confounding population structure in half of the strains sampled [14,15]. The other half is represented by the mosaics strains and may be more suitable for GWAS. However, the linkage disequilibrium blocks in mosaics strains appear large (hundreds of kilobases); thus they retain local substructure and risk a high false positive rate. Optimal GWAS experiments, in yeast, will require both better sampling and understanding of yeast population structure. Computational approaches exist to control for and stratify the population structure [70–72]. Further population genomics analysis will be informative on whether the budding yeast is a suitable model for GWAS. Given the thousands of strains now available, GWAS may in the future prove to be applicable to yeast. Alternatively, populations of wild yeast strains can be manipulated to force rounds of mating and sporulation to create synthetic outbreed populations for association mapping as previously used in *Drosophila melanogaster* [73]. A common problem of GWAS is the ability to detect functional polymorphisms that occur at low allele frequency in a population [74]. The QTLs identified so far indicated that many of the variants with large effect are rare, although a much larger sample is required to determine overall allele frequency. It is unclear whether or not traits are generally determined by rare large effect QTLs or if these are just more readily detected due to their large effect. Furthermore, it will be interesting to understand when these large effect polymorphisms arose since it remains a possibility that they were acquired within the laboratory setting [58].

## 4 Bridging the sequence to function gap using multiple approaches

### 4.1 Telomere length

In this section, we illustrate how a combination of genomic approaches has contributed to our knowledge of a highly complex phenotype, telomere length homeostasis. This trait is of primary medical importance due to its central role in genome stability with impact for both cancer and ageing [75]. Telomere length has been extensively investigated by classical cell biology methods. More recently, new insights have been gleaned from reverse genomics and by exploiting the natural variation of related species and strains.

Most eukaryotic chromosomes end with species specific G-rich DNA repeats. These repeats are maintained and protected by a number of protein complexes and are crucial to genome stability, cancer and ageing. In *S. cerevisiae* telomeres consist of ~ 350 bp of degenerated TG_1-3_ repeats. This average length is maintained as the result of telomere attrition due to incomplete end replication and extension due to the activity of the specialised reverse transcriptase, telomerase. Yeast has played a key role in understanding the molecular mechanisms that regulate telomere length homeostasis [76].

Comparative genomics of different chromosome ends helped to map conserved motifs such as the core X element, protein binding sites (e.g. Abf1p) and replication origins [76]. The mapping of these units provided important insights into the regulation of key telomeric properties such as length, transcriptional silencing, recombination and replication. Sequence comparison has been also used to characterise trans-acting telomere regulator. The secondary structure of the RNA template of telomerase, *TLC1*, could not be experimentally determined due its complexity and large size. The analysis of compensatory mutations (covariation) occurring through evolution allowed the identification of conserved RNA helices and thus the deduction of the overall secondary structure [77,78].

Two reverse genomics studies have screened the entire gene deletion collection for telomere length maintenance (TLM) genes [79,80]. These studies produced overlapping, but not identical, lists of TLM genes, illustrating the challenge of using the deletion collection to study a complex phenotype. An additional study screened for TLM genes in a library of essential gene mutants with altered gene expression [81]. The TLM genes span a broad range of functions and their effect is either telomerase-dependent or independent. Collectively, these studies revealed 361 TLM genes indicating that this trait is affected by a large fraction of the gene repertoire (6.5% of total ORFs). This number is likely to increase through computational predictions [82] or if the screening is extended to different strain backgrounds [39]. Indeed, deletion of several TLM genes in different *S. paradoxus* strains revealed a dramatic effect of the genetic background even for key telomeric protein components such as *yKu70* and *yKu80* [83]. Furthermore, telomere length differences between two laboratory strains appear to be responsible for the requirement for *RAD52* in telomerase negative mutants [42,43]. A recent study reports the screening of a *Schiz. pombe* deletion collection for TLM genes, but due to the incomplete nature of the collection it is currently difficult to make comparisons with the *S. cerevisiae* TLM genes [84]. Surprisingly and in direct contrast to *S. cerevisiae*, the majority of *Schiz. pombe* TLM gene deletions result in longer telomeres. In summary, the screening of yeast deletion collections has identified many novel genes involved in telomere length regulation.

Screening of telomere length in natural populations of *S. cerevisiae* and *S. paradoxus* revealed extensive variation [83]. This has allowed investigation of telomere length regulation using forward genomics and the detection of multiple QTLs [80,83]. Two of the detected QTL regions contain previously identified TLM genes and have been experimentally validated [83]. This suggests that the TLM genes identified by functional analysis are also responsible for variation in natural population. Furthermore, one strong QTL was mapped within a region with no previously reported TLM genes demonstrating that linkage analysis can be used to detect novel regulators. Further characterisation of telomere length as a complex trait will be possible, including in combination with chemical genetic screens [85], to understand the impact of environmental conditions and their interaction with the genetic background (Fig. 1b).

## 5 Conclusions

Establishing how genotype determines phenotype represents the next frontier in biomedical science. Achieving this goal will require an understanding of the quantitative differences between individuals, for example the response to environmental risk factors or medical treatments. In yeast, high throughput phenotyping (phenomics) has allowed the characterisation of many strains, both natural isolates [14,86] and deletion collections [31]. However, dissecting the genetic mechanisms underlying phenotypic variation represents a major challenge. The budding yeast, *S. cerevisiae*, has helped with this daunting task in two ways. First, knowledge gained about individual genes in *S. cerevisiae* can frequently be directly extrapolated to metazoan orthologs. Second, yeast has been the test bed for many genome-wide systems biology approaches, subsequently implemented in other organisms.

Classical studies have used both forward and reverse genetic approaches to dissect multiple phenotypes in the laboratory strain S288c and its derivatives. Recently, there has been an increased appreciation for dissecting the phenotypic variation within natural populations with the goal of revealing the genetic structure of a trait. Both reverse and forward genomic approaches have been applied to natural populations and each has their strengths. Genetic and genomic approaches are limited to studying inherited traits and their analysis is complicated by traits that are largely determined by the environment. Forward genomic approaches offer the advantage of investigating functional variants in a specific genetic context and are therefore not limited to looking at particular genes. Furthermore, they have the ability to investigate essential genes, which still present difficulties for reverse genomic approaches. On the other hand, forward genomic approaches are limited by the extent of natural variation, although it is worth noting that even in the absence of phenotypic variation between parental strains there can be extensive variation between segregants [56]. Additionally, the natural variation in a specific trait can be limited to a small fraction of genes that make up the specific trait. By contrast, reverse genomics potentially has the ability to detect all the genes present in a pathway. However, reverse genomic approaches also have shortcomings; for example gene deletions poorly reflect natural evolution, the deletion cassettes may affect neighbouring genes (creating false positives) and secondary mutations may have arisen during laboratory manipulations. Finally, reverse genomic approaches cannot predict the individual quantitative phenotypic difference from a given genotype. Combining comparative genomics and functional analysis (reverse genomics) with QTL mapping (forward genomics) has the potential to uncover the broad structure of a trait [63,87]. Understanding the principles by which genotype determines phenotype in yeast will facilitate the development of predictory models [51], which in the future could be applied to understanding human disease.

## Figures and Tables

**Table 1:** Useful websites.

Hemiascomycetes genome sequence databases	
Yeast Genome Database	http://www.yeastgenome.org/
Broad Institute Yeast Comparative Genomics	http://www.broadinstitute.org/annotation/fungi/comp_yeasts/index.html
UCSC Genome Browser	http://genome.ucsc.edu/
Génolevures—hemiascomycete yeasts	http://www.genolevures.org/
Washington University *Saccharomyces* Genomes	http://www.genetics.wustl.edu/saccharomycesgenomes/
*Ashbya* Genome Database	http://agd.vital-it.ch/index.html
*Candida* Genome Database	http://www.candidagenome.org/


*S. cerevisiae* population genomic databases	

*Saccharomyces* Genome Resequencing Project	http://www.sanger.ac.uk/research/projects/genomeinformatics/sgrp.html
*Saccharomyces cerevisiae* Strain Project	http://genome.wustl.edu/genomes/list/saccharomyces_cerevisiae_strain_project/
Yeast SNPs Browser	http://gbrowse.princeton.edu/cgi-bin/gbrowse/yeast_strains_snps/
